# Fluid overload after coronary artery bypass graft in patients on maintenance hemodialysis is associated with prolonged time on mechanical ventilation

**DOI:** 10.1186/s12871-020-00971-6

**Published:** 2020-03-07

**Authors:** Sirlei Cristina da Silva, Fernanda Marciano Consolim-Colombo, Renata Gomes Rodrigues, Fábio Antonio Gaiotto, Ludhmila Abrahão Hajjar, Rosa Maria Affonso Moysés, Rosilene Motta Elias

**Affiliations:** 1 Instituto do Coração, Hospital das Clinicas HCFMUSP, Universidade de São Paulo, São Paulo, Brazil; 2 Nephrology, Hospital das Clinicas HCFMUSP, Universidade de São Paulo, São Paulo, Brazil; 3 Universidade Nove de Julho (UNINOVE), São Paulo, Brazil

**Keywords:** Hemodialysis, Intensive care unit, Renal disease, Dialysis, Chronic kidney disease

## Abstract

**Background:**

Fluid overload is a risk factor for morbidity, mortality, and prolonged ventilation time after surgery. Patients on maintenance hemodialysis might be at higher risk. We hypothesized that fluid accumulation would be directly associated with extended ventilation time in patients on hemodialysis, as compared to patients with chronic kidney disease not on dialysis (CKD3–4) and patients with normal renal function (reference group).

**Methods:**

This is a prospective observational study that included patients submitted to isolated and elective coronary artery bypass surgery, divided in 3 groups according to time on mechanical ventilation: < 24 h, 24-48 h and > 48 h. The same observer followed patients daily from the surgery to the hospital discharge. Cumulative fluid balance was defined as the sum of daily fluid balance over the first 5 days following surgery.

**Results:**

Patients requiring more than 48 h of ventilation (5.3%) had a lower estimated glomerular filtration rate, were more likely to be on maintenance dialysis, had longer anesthesia time, needed higher dobutamine and noradrenaline infusion following surgery, and had longer hospitalization stay. Multivariate analysis revealed that the fluid accumulation, scores of sequential organ failure assessment in the day following surgery, and the renal function (normal, chronic kidney disease not on dialysis and maintenance hemodialysis) were independently associated with time in mechanical ventilation. Among patients on hemodialysis, the time from the surgery to the first hemodialysis session also accounted for the time on mechanical ventilation.

**Conclusions:**

Fluid accumulation is an important risk factor for lengthening mechanical ventilation, particularly in patients on hemodialysis. Future studies are warranted to address the ideal timing for initiating dialysis in this scenario in an attempt to reduce fluid accumulation and avoid prolonged ventilation time and hospital stay.

## Background

Coronary artery bypass grafting (CABG) is indicated as a treatment of ischemic heart disease for patients with chronic kidney disease [1] (CKD), a population with a high mortality rate. Respiratory failure is common during the postoperative period following CABG and continues to be a major cause of morbidity in this population [2, 3]. Mechanical ventilation in the postoperative period is needed until normothermia and hemodynamic stability is achieved [4]. Intubation time is the strongest independent predictor of 30-day and 1-year mortality among patients undergoing CABG [5]. Modern surgical techniques, advances in anesthesia and myocardial protection have contributed to reducing the ventilation time, which is increased by age and comorbidities [2]. Prolonged mechanical ventilation (PMV) has been described in 2.9 to 22% of patients submitted to CABG [2, 6]. The first 24 h of mechanical ventilation are dependent on multiple factors, including a patient’s preoperative condition, the complexity of surgical procedure, as well as intra- and postoperative complications [5].

Hemodynamic instability after cardiovascular surgery is a situation often managed with fluid administration. However, establishing goals of volume management in patients with renal failure on maintenance hemodialysis is challenging. Since these patients are usually anuric, fluid accumulation is not uncommon. The association between positive fluid balance and deleterious effects on lung function and prolonged mechanical ventilation has been described [7, 8]. Indeed, positive fluid balance during the first 3 to 7 days can increase in-hospital mortality even in non-cardiac, postsurgical patients [9]. Negative fluid balance, on the other hand, is associated with lower postoperative mortality following both cardiovascular surgery [8, 10] and non-cardiovascular surgery [7].

Anuria and the high prevalence of comorbidities such as hypertension, diabetes and advanced age increase the odds of a positive fluid balance, and PMV in these patients [11]. The goal of the current study is to access the time on mechanical ventilation after CABG, comparing patients with normal renal function, patients with CKD not on dialysis, and patients on regular maintenance hemodialysis. We hypothesized that patients on dialysis will present a more positive fluid balance and, therefore, prolonged time on mechanical ventilation.

## Methods

**Fig. 1:**
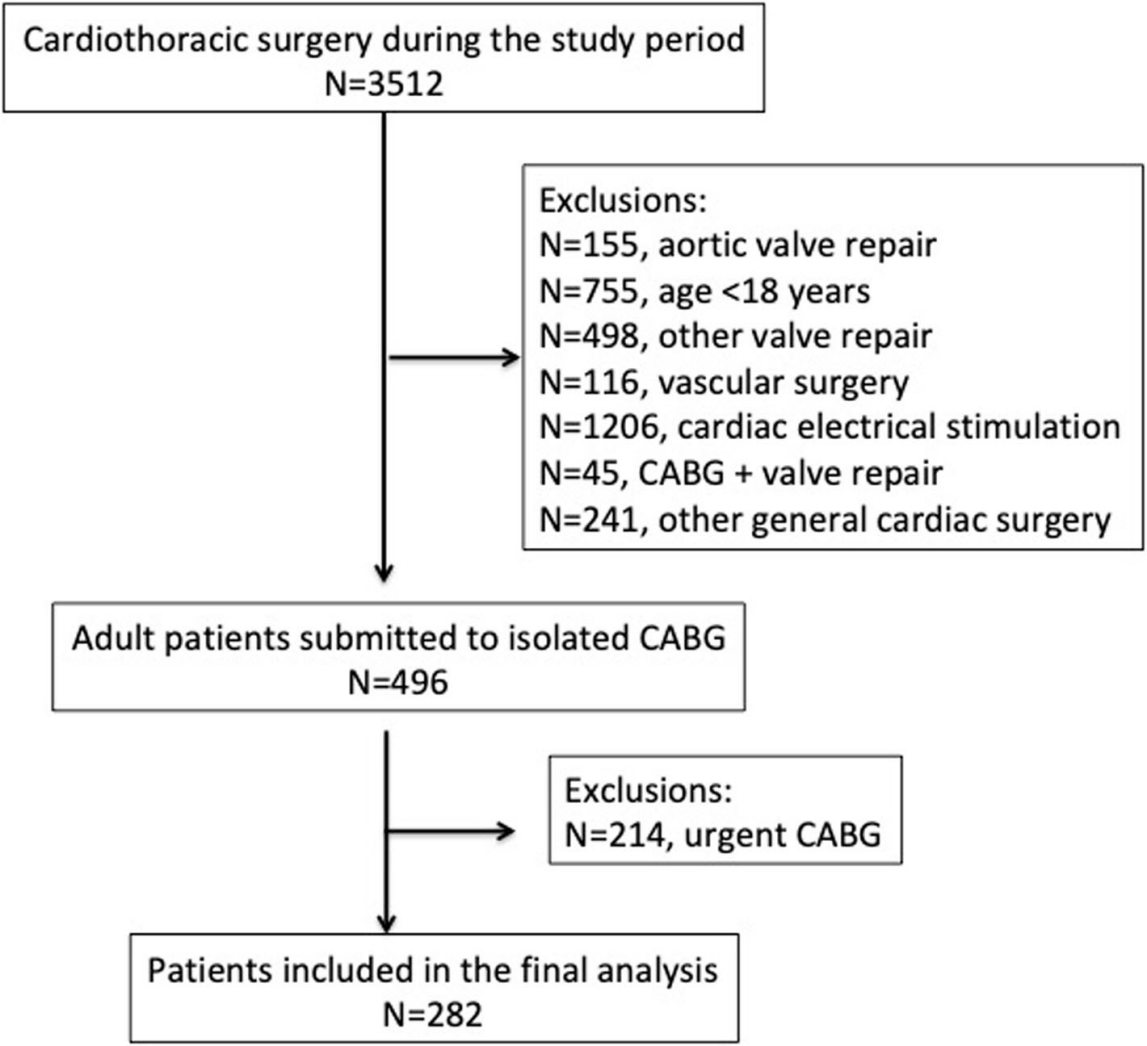
Flow diagram for patient inclusion and exclusion

Patients were recruited at the Instituto do Coração (InCor), Universidade de São Paulo. Inclusion criteria were as follow: consecutive adult patients submitted to an elective CABG in the period between July 2015 and March 2017. Flow diagram for patient inclusion and exclusion is shown in a supplementary file (Fig. 1). For analysis purpose patients were fitted according to mechanical ventilation length after surgery (less than 24 h, 24–48 h and more than 48 h). The exclusion criterion was patients submitted to valve replacement surgery plus CABG.

The Local Ethics Committee at the Hospital das Clínicas da Faculdade de Medicina da Universidade de São Paulo has approved the research (Cappesq #45529815.6.0000.0068).

### Variables of interest and definitions

Clinical, biochemical and demographic data were prospectively collected from charts including age, gender, weight, presence of diabetes, and serum creatinine. Postoperative data collected included: aortic cross-clamping time (min), cardiopulmonary bypass time (min), surgery time (min), use of intra-aortic balloon pump (% of patients), anesthesia time (min), ventilation time (also categorized in < 24, 24-48 h and > 48 h), sequential organ failure assessment - SOFA (scores) and use of dobutamine and noradrenaline.

Renal function was expressed as estimated glomerular filtration rate (eGFR), calculated by the Chronic Kidney Disease Epidemiology - CKD-EPI 2009 equation [12]. Patients on renal replacement therapy were submitted to a hemodialysis session on the day before surgery, according to Hospital protocol.

The same observer followed each patient daily from the surgery to the hospital discharge. To identify the potential risk of mortality, the SOFA score was applied. In addition, we calculated the same score without taking into account the renal component. Daily fluid balance was calculated during intensive care unit (ICU) as the difference in intakes and outputs, not including insensible losses, taking into account: volume of fluid intake (including saline, drugs and blood), and losses (ultrafiltration during hemodialysis, diuresis, and blood loss, quantified as volume drained in the thoracic suction tube) [13].

A positive balance defined fluid accumulation. Cumulative fluid balance was defined as the sum of daily fluid over the first 5 days after CABG (Σ fluid balance). Fluid overload (FO) was defined as 10% after adjustment for body weight (FO/body weight) and it was calculated as following: % fluid overload = (total fluid in - total fluid out)/admission body weight × 100), expressed as percentage [13].

### Statistical analysis

Continuous data are expressed as mean ± standard deviation (SD) or median (25, 75), whereas categorical data are expressed as frequencies and percentages. Comparison among the 3 groups was done by ANOVA (if normally distributed) or Kruskall-Wallis (if non-normally distributed). Categorical data were compared by Fisher’s exact test or chi-squared, as appropriate. Relationships between single variables were examined by Spearman. Multivariate regression analyses were used to assess factors associated with ventilation time and independent variables were selected from univariate analysis. We also performed a stepwise linear regression, with *p* < 0.05 to enter and *p* > 0.1 to remove in the group of patients on hemodialysis to test age, SOFA scores without the renal component, and the accumulated fluid balance (Σ fluid balance). Analyses were performed with the use of SPSS 22.0 (SPSS Inc., Chicago, IL) and GraphPad® Prism 8.0 (GrapPad Software Inc., San Diego, CA, USA). Two-sided *P* values < 0.05 were considered statistically significant.

## Results

In the study population, 77.3% of patients did not require ventilation for more than 24 h, while 17.4 and 5.3% were on mechanical ventilation 24–48 h and > 48 h, respectively. Baseline characteristics of patients according to time on mechanical ventilation are shown in Table 1. Patients requiring more than 48 h of ventilation had a lower eGFR, were more likely to be on maintenance dialysis and had similar SOFA at the ICU admission, not taking into account the renal component. Intraoperative condition that differed patients on prolonged ventilation (> 48 h) were the longer anesthesia time, the higher dobutamine and noradrenaline dosage during 24 h following CABG, and longer hospitalization and ICU stay (Table 1).

**Table 1:** Patient baseline characteristics, according to time on mechanical ventilation

Baseline characteristics	Less than 24 h ***N*** = 218	24–48 h ***N*** = 49	More than 48 h ***N*** = 15	***p***
**Age, years**	63 ± 9	62 ± 8	64 ± 9	0.844
**Weight, kg**	76 ± 14	77 ± 13	72 ± 9	0.577
**Male gender, %**	72.5	81.6	80	0.364
**Diabetes, %**	52.3	61.2	46.7	0.453
**Ejection fraction, %**	55.9 ± 11.4	52.7 ± 15.2	56.5 ± 11.6	0.307
**eGFR, ml/min/1.73 m**^**2**^	69.2 ± 18.6 ^**†**^	63.8 ± 19.8 ^**†**^	44.2 ± 14.8*	**0.0001**
**Serum creatinine at admission, mg/dl**	1.13 ± 0.4 ^**†**^	1.26 ± 0.5 ^**†**^	1.69 ± 0.8*	**0.0001**
**Patients, from each group %**				**0.0001**
** Control (N = 167)**	83.8	15.6	0.6	
** CKD3–4 (N = 84)**	67.9	21.4	10.7	
**Dialysis (*N*** **= 31)**	67.7	16.1	16.1	
**Surgery and ICU conditions**				
**ACC time, min**	69.1 ± 28.9	72.6 ± 26.0	69.6 ± 20.1	0.758
**CPB time, min**	92.1 ± 35.9	93.2 ± 30.3	95.5 ± 14.7	0.932
**Surgery time, min**	383 ± 97	377 ± 115	433 ± 173	0.173
**Diuresis IPO, ml/kg/h**	0.73 ± 0.35	0.70 ± 0.38	0.57 ± 0.63	0.274
**Diuresis 1st day after surgery, ml/kg/h**	1.18 ± 0.46	1.18 ± 0.49	0.83 ± 0.77*	**0.035**
**Intra-aortic balloon pump, %**	4.2	7.1	0	0.248
**Fluid balance 24 h after surgery, L**	2.5 ± 1.2	2.7 ± 1.2	2.7 ± 1.3	0.749
**Anesthesia time, min**	412 ± 86 ^**†**^	417 ± 98 ^**†**^	482 ± 164*	**0.021**
**Ventilation time, hours**	8 (6, 10) ^**†**^	16 (14, 19)*^†^	62 (42, 160)*	**0.0001**
**SOFA on the ICU admission**	0 (0, 1) ^**†**^	1 (0, 1.22) ^**†**^	3 (1, 4)*	**0.0001**
**SOFA without renal component**	0 (0, 0)	0 (0, 1)	0 (0, 1)	0.326
**SOFA by organ**				
**Renal**	0 (0, 1)	0 (0, 1) ^**†**^	1 (1, 4)*	**0.0001**
**Hematologic**	0 (0, 0)	0 (0, 0)	0 (0, 1)	0.106
**Neurologic**	0 (0, 0)	0 (0, 0)	0 (0, 0)	1
**Respiratory**	0 (0, 0)	0 (0, 0)	0 (0, 0)	0.630
**Hepatic**	0 (0, 0)	0 (0, 0)	0 (0, 0)	0.863
**Cardiovascular**	0 (0, 0)	0 (0, 0)	0 (0, 1)	0.092
**Dobutamine dose 24 h after surgery, ml/kg/min**	7.5 ± 5.0 ^**†**^	9.9 ± 5.5*^†^	12.4 ± 6.0*	**0.0001**
**Noradrenaline dose 24 h after surgery ml/kg/h**	0.16 ± 0.14 ^**†**^	0.27 ± 0.19*^†^	0.56 ± 0.13*	**0.0001**
**Volume intake in 5 days, L**	7.6 ± 1.9	7.3 ± 1.8	8.1 ± 0.9	0.725
**Cumulative fluid balance in 5 days, L**	−1.3 (−2.4, −0.3)	−1.6 (−2.6, 0.3)	0.39 (−2.7, 3.7)	0.109
**Σ Fluid balance, L**	1.2 (−0.4, 2.4)	1.0 (−1.2, 2.3)	1.8 (0.9, 7.8)	0.208
**Fluid overload/body weight, %**	−1.9 (−3.0, −0.5)	−1.8 (−3.5, 0.5)	0.6 (−3.4, 5.6)	0.109
**Hospitalization time, days**	14 (10, 20)	16 (12, 29)* ^**†**^	23 (17, 43)*	**0.001**
**ICU stay, days**	3 (2, 5) ^**†**^	5 (3, 7)* ^**†**^	8 (5, 27)*	**0.0001**

Data are presented as mean SD, % or median (25–75)

*ACC time* aortic cross-clamping time, *CPB time* cardiopulmonary bypass time, *SOFA* Sequential Organ Failure Assessment*, ICU* intensive care unit

* *p* < 0.05 vs. Less than 24 h; **†***p* < 0.05 vs. More than 48 h

Five patients (all from the CKD3–4 group) developed an impairment of renal function and required dialysis during hospitalization. These patients were characterized by higher serum creatinine (*p* = 0.045) and SOFA scores upon admission (*p* = 0.035) than those from the same group that did not required dialysis.

**Fig. 2:**
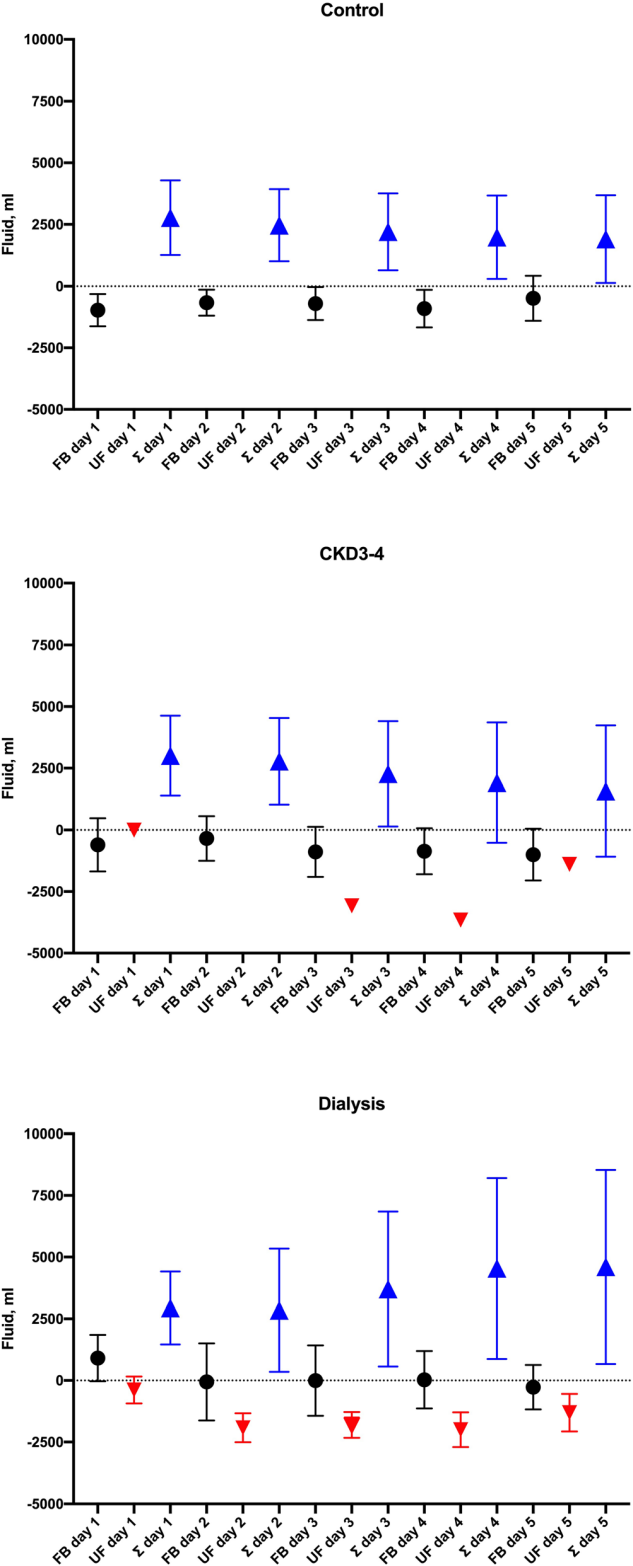
Fluid accumulation in the 5 days following coronary artery bypass surgery according to renal function. Daily fluid balance (result of intake and output) is represented by a dark circle. Ultrafiltration promoted by dialysis is represented by a red triangle, and Σ fluid balance (cumulative result of intake and output) is represented by a blue triangle. Of note, patients with normal renal function (reference – upper panel) were capable to maintain fluid balance close to zero. Patients with stages 3–4 chronic kidney disease – middle panel) presented a slightly positive fluid balance and some of them needed dialysis due to acute renal failure. Patients on maintenance hemodialysis (bottom panel) exhibited a positive and cumulative fluid balance despite an ultrafiltration promoted by dialysis

Although there was no difference in fluid balance during the first 24 h after surgery, patients on maintenance hemodialysis had a more positive fluid accumulation 48 h after CABG, even considering the negative balance promoted by ultrafiltration, as depicted in Fig. 2.

**Fig. 3:**
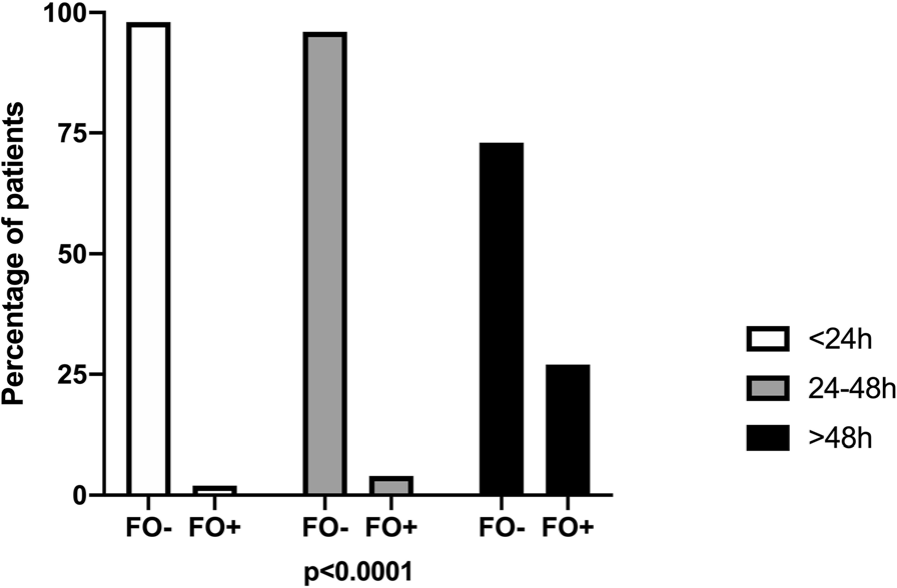
Association between fluid overload (FO) and time on mechanical ventilation. Patients on mechanical ventilation for < 24 h, 24-48 h and > 48 h were represented by white, grey and black bars, respectively

We found than only 10 patients presented FO (> 10%). FO was found in 4 (1.8%), 2 (4.1%) and 4 (26.7%) patients on mechanical ventilation for < 24 h, 24–48 and > 48 h, respectively (*p* = 0.001), as shown in Fig. 3. In addition, patients with FO > 10% were more likely to be on maintenance hemodialysis (16.1% on hemodialysis vs. 3.6% of patients with CKD not on dialysis and 1.2% of patients with normal renal function, *p* = 0.0001).

Ventilation time correlated with eGFR (*r* = − 0.183, *p* = 0.004), SOFA at admission (*r* = 0.185, *p* = 0.002) and on the first day after surgery with and without the renal component (*r* = 0.482, *p* = 0.0001 and *r* = 0.505, *p* = 0.0001, respectively), hospitalization time (*r* = 0.230, *p* = 0.0001) and ICU stay (*r* = 0.326, *p* = 0.0001). There was no significant association between time on mechanical ventilation and pneumonia (*p* = 0.389), diabetes (*p* = 0.453), hypertension (*p* = 0.752), dyslipidemia (*p* = 0.373), obesity (*p* = 0.624), history of previous cardiac surgery (*p* = 0.464), ischemic cardiomyopathy (*p* = 0.718), history of previous myocardium infarction (*p* = 0.874), history of cancer (*p* = 0.372), urinary infection (*p* = 0.843), and operative site infection (*p* = 0.105).

In a multivariate analysis, factors found to be independently associated with time ventilation time were the Σ fluid balance (*p* = 0.011), group of patients (*p* = 0.039), and the SOFA on the first day after surgery (*p* = 0.0001), in a model adjusted for anesthesia time, noradrenaline and dobutamine dosage (Table 2).

**Table 2:** Multivariate analysis of factors associated with prolonged time on ventilation in the entire population and among patients on maintenance hemodialysis

Parameter	Standardized β coefficient	Partial correlation	***p***
**Model 1: Entire population**			
Σ fluid balance, ml	0.171	0.172	0.004
Group of patients: reference, CKD3–4 and dialysis	0.182	0.183	0.002
SOFA on the first day after surgery	0.312	0.314	0.0001
**Model 2: Patients on hemodialysis**			
Σ fluid balance, ml	0.284	0.497	0.049
Time from surgery to 1st HD, h	0.289	0.332	0.038
SOFA without renal component	0.549	0.627	0.0001

Model 1: *r* = 0.369, *r*^2^ = 0.136 and adjusted *r*^2^ = 0.130; *p* = 0.0001. Other variables in the model: anesthesia time, noradrenaline and dobutamine dose 24 h after surgery

Model 2: *r* = 0.757, *r*^2^ = 0.574 and adjusted *r*^2^ = 0.524; *p* = 0.0001. Other variable in the model: age

*HD* hemodialysis, *SOFA* Sequential Organ Failure Assessment

We further performed a multivariate analysis including only patients on maintenance hemodialysis; the time on mechanical ventilation was dependent on the Σ fluid balance and the SOFA on the first day after surgery (without the renal component) that together accounted for 52.4% in the variability of the time on mechanical ventilation (Table 2).

## Discussion

Fluid overload in patients on dialysis is a therapeutic challenge as it can lead to several unfavourable outcomes [14]. In this prospective study, we made the novel observation that fluid accumulation was directly associated with prolonged mechanical ventilation in patients in this population. We also observed that the time spent since the CABG until the first hemodialysis session was another independent predictor factor of prolonged ventilation. Whether early dialysis would change this scenario warrants further studies.

PMV has been associated with fluid overload. In the present study, patients who required more than 48 h of ventilation had lower eGFR and most of them were from the dialysis group. The propensity to vascular congestion and alveolar volume overload in patients with end-stage renal helps justify these data [15]. Canver et al. showed that patients with renal failure had 12.8 odds to develop respiratory failure [2]. Even in patients with normal renal function, fluid overload is associated with extravasation into the interstitial space and reduction of capillary blood flow leading to renal ischemia [8, 10].

A previous prospective study has shown that progressive fluid overload and changes in creatinine correlated with post-cardiac surgery mortality [16]. Indeed, fluid overload was associated with prolonged length in ICU and it was identified as an earlier and more sensitive prognostic marker than serum creatinine [16]. Heringlake et al. in a post-hoc study enrolling 584 patients showed that 7.4% of patients developed AKI stage 3 and initiated dialysis 26.5 h after surgery [4]. The early initiation of dialysis showed a survival advantage for this population. However, the ideal moment to initiate dialysis is controversial, and there is opposition to early dialysis because it could expose patients to potential harms such as intradialytic hypotension [15]. Chronic or acute functional changes at the renal system were associated with failure or delayed extubation in clinical and surgical patients [6, 17]. It is possible to perceive the narrow relationship between the renal and pulmonary system and unclear unrecognized risk factors, which need to be explored.

Despite ultrafiltration during the hospitalization stay, patients on dialysis developed FO, and can cause extravasation of fluid into interstitial space, increasing extravascular lung water, decreasing lung compliance and impairing oxygenation, which results in respiratory failure and impairment of multiple organ systems [8, 10, 17]. Our study showed an association between fluid accumulation, ICU stay and ventilation time. Fluid accumulation became significant after 24 h post-operative, which was remarkable in patients with CKD3–4 and in those on dialysis. In a retrospective study that enrolled 567 patients submitted to cardiovascular surgery, the delay to reach a negative fluid balance during the first 3 days was associated with higher hospital length of stay and mortality [10]. Our data showed that patients with normal renal function had an effective homeostasis mechanism that promotes negative balance. However, some patients with CKD3–4 had a progressive fluid accumulation and needed dialysis. In patients on maintenance hemodialysis, this scenario was worse as FO persisted despite consecutive ultrafiltration, measured by Σ fluid accumulation. The high amplitude fluctuation in the fluid has been related to 2.75 times higher all-cause and cardiovascular mortality in patients on maintenance hemodialysis [14]. The hemodynamic instability after CABG despite the fluid overload might postpone the decision to initiate dialysis in the clinical practice [16]. Nevertheless, based on our findings, fluid accumulation correlated with ventilation time in patients on dialysis, which denotes the importance of hemodialysis in this group. Σ fluid accumulation was independently associated with prolonged time on mechanical ventilation. Moreover, the longer the time spent to initiate the first dialysis session, the longer the ventilation time.

Our results denote that fluid accumulation is a marker of prolonged ventilation in patients on maintenance hemodialysis submitted to an elective CABG. Therefore, our study opens an avenue for research on the ideal time to initiate dialysis after such surgery, in an attempt to reduce fluid accumulation and avoid extending ventilation time.

This study is subject to some limitations: first, the acid-base equilibrium was not analyzed; second, the moment to initiate dialysis was dependent on the physician in charge; third, the daily weight was not available; fourth, due to a limited sample size (*N* = 5) we could not adjust for acute renal failure that occurred in the CKD3–4 group, and finally, due to the study design we were not able to access if early dialysis initiation would short the time on mechanical ventilation. The strength of our study was its prospective design and the daily follow-up by the same observer.

## Conclusions

Our findings suggest that prolonged ventilation time in patients on maintenance hemodialysis might be directly dependent on the fluid overload and the time spent until the first hemodialysis session.

## Data Availability

The datasets analysed during the current study are available from the corresponding author on reasonable request.

## Abbreviations

CABG: Coronary artery bypass grafting
CKD: Chronic kidney disease
eGFR: Estimated glomerular filtration rate
ICU: Intensive care unit
PMV: Prolonged mechanical ventilation
SOFA: Sequential organ failure assessment
Σ: Sum of fluid balance
